# Analytic Approaches in Genomic Epidemiological Studies of Parasitic Protozoa

**DOI:** 10.1155/2024/7679727

**Published:** 2024-06-08

**Authors:** Tianpeng Wang, Ziding Zhang, Yaoyu Feng, Lihua Xiao

**Affiliations:** ^1^ State Key Laboratory for Animal Disease Control and Prevention South China Agricultural University Guangzhou 510642China; ^2^ Guangdong Provincial Key Laboratory of Utilization and Conservation of Food and Medicinal Resources in Northern Region Shaoguan University Shaoguan 512005China; ^3^ State Key Laboratory of Animal Biotech Breeding College of Biological Sciences China Agricultural University Beijing 100193China; ^4^ Guangdong Laboratory for Lingnan Modern Agriculture Guangzhou 510642China

## Abstract

Whole genome sequencing (WGS) plays an important role in the advanced characterization of pathogen transmission and is widely used in studies of major bacterial and viral diseases. Although protozoan parasites cause serious diseases in humans and animals, WGS data on them are relatively scarce due to the large genomes and lack of cultivation techniques for some. In this review, we have illustrated bioinformatic analyses of WGS data and their applications in studies of the genomic epidemiology of apicomplexan parasites. WGS has been used in outbreak detection and investigation, studies of pathogen transmission and evolution, and drug resistance surveillance and tracking. However, comparative analysis of parasite WGS data is still in its infancy, and available WGS data are mainly from a few genera of major public health importance, such as *Plasmodium*, *Toxoplasma*, and *Cryptosporidium*. In addition, the utility of third-generation sequencing technology for complete genome assembly at the chromosome level, studies of the biological significance of structural genomic variation, and molecular surveillance of pathogens has not been fully exploited. These issues require large-scale WGS of various protozoan parasites of public health and veterinary importance using both second- and third-generation sequencing technologies.

## 1. Introduction

The advent of new sequencing technologies has greatly promoted pathogen characterization, bringing pathogen research into the era of precision public health [1]. With the high throughput and low cost of next-generation sequencing (NGS), whole genome sequencing (WGS) has been widely applied to investigate the epidemiology of major infectious diseases, such as tuberculosis [2], foodborne outbreaks of Shiga toxin-producing *Escherichia coli* O157:H7 [3], and newly emerged COVID-19 [4]. The development of third-generation sequencing (TGS) technologies with long sequence reads has overcome challenges in assembling the full genomes of eukaryotic pathogens for comparative analysis of genome structural variations [5]. Among them, nanopore sequencing-based portable sequencing technique has been established for molecular surveillance of some epidemic diseases [6]. In addition, the chromatin conformation capture coupled with NGS (Hi–C) is another promising method that can provide an unbiased all-by-all genome-wide interaction map, which can help not only to improve the contiguity of assembly from the NGS and TGS data [7] but also to describe the 3D genome structure [8]. In general, WGS-based pathogen characterizations provide high-resolution pathogen differentiation, enabling accurate case linkage and infection source tracking during outbreak investigations. They also shed lights on pathogen emergence, dispersal, and evolution, promoting understanding of the transmission dynamics of emerging and zoonotic pathogens [9].

Protozoan parasites are unicellular eukaryotes causing mostly hematological, neurological, cardiac, and gastrointestinal diseases in both humans and livestock. Apicomplexans are a large group of protozoa with a unique invasion complex, including *Plasmodium* spp., *Babesia* spp., *Theileria* spp., *Toxoplasma gondii*, *Sarcocystis* spp., *Cystoisospora* spp., *Cyclospora cayetanensis*, *Eimeria* spp., and *Cryptosporidium* spp. They cause several diseases of major public health and veterinary importance, including malaria, piroplasmosis, toxoplasmosis, coccidiosis, and cryptosporidiosis. The global burden of some parasitic diseases has increased significantly with the rise of intensive animal farming, international travel and trade, global migration, and climate and environmental changes [10]. Only a small number of drugs are available for some of the parasitic diseases. The development of new antiparasitic drugs is facilitated by WGS of the pathogens, allowing more rapid identification of drug targets based on comparative analyses of metabolism pathways and whole-genome profiling of chemically induced mutants [11]. As natural selection may result in increased occurrence of drug resistance, the availability of WGS data would allow accurate identification and tracking of drug resistance in parasitic pathogens [12]. These WGS data also improve our knowledge of the epidemiology of parasitic diseases and guide us in the development of control measures and pandemic preparedness through advanced tracking of the dispersal and evolution of pathogens [13].

In this review, we aim at discussing the utility of WGS in our understanding of the genomic epidemiology of apicomplexan parasites with high prevalence and major public health importance. We have outlined the utility of WGS data, described some major bioinformatics approaches used in data analyses, and discussed the need for large-scale WGS of diverse field isolates for the development of WGS-based surveillance systems.

## 2. Overview of WGS Data on Apicomplexan Parasites

Currently, large genome datasets are available for a few protozoan parasites of major public health and economic importance. Genomes were traditionally sequenced using the Sanger-sequencing technology, with the complete genome of *Plasmodium falciparum* being the first one published in 2002, which is 23.3 Mb in 14 chromosomes and has a high AT ratio (80.7%) [14]. This was followed by genomes of *Cryptosporidium parvum* and *Cryptosporidium hominis*, which were published in 2004 [15, 16] and the genome of *Toxoplasma* released in 2005 [17]. The WGS of apicomplexans is greatly facilitated by the development of NGS technologies and more recently by the TGS technologies. There are currently 359 apicomplexan genomes available in the NCBI Genome Datasets (https://www.ncbi.nlm.nih.gov/data-hub/genome/) by searching for “Apicomplexa” (accessed on May 3, 2023), including 85 reference genomes. In VEuPathDB (https://veupathdb.org/veupathdb/app), a one-for-all genomic resource for eukaryotic pathogens and invertebrate vectors, 143 assemblies of apicomplexan genomes including 70 reference genomes are available (accessed on May 3, 2023). Curated reference genomes are available in pathogen-specific databases such as PlasmoDB (https://plasmodb.org/plasmo/app), ToxoDB (https://toxodb.org/toxo/app), and CryptoDB (https://cryptodb.org/cryptodb/app). More WGS data without assembled genomes are available from the NCBI Sequence Read Archive (SRA) database.

**Figure 1:**
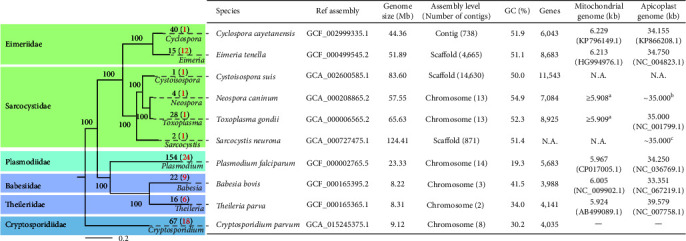
Phylogenic relationship and genome statistics of major apicomplexan species. Reference genomes of 10 apicomplexan genera were downloaded from NCBI. The rooted maximum likelihood (ML) tree was constructed with 288 single-copy genes, with *Leishmania major* as the outgroup (not shown). Single copy genes were extracted using Orthofinder v2.5.4 [18]. The ML tree was constructed with IQ-TREE v2.1.2 [19] with a bootstrap value 1,000 and the substitution model automatically selected with *ModelFinder Plus* (MFP). The number at each tip represents the number of published genomes, with number of reference genomes in parentheses. Genome statistics were mainly referred to NCBI datasets (https://www.ncbi.nlm.nih.gov/datasets/, accessed on May 3, 2023). However, the numbers of chromosomes in *Toxoplasma gondii* and *Neospora caninum* have been updated according to recent genomic studies [5, 20]. N.A., no information available. There is no such organelle in *Cryptosporidium*. ^a^Namasivayam et al. [21]; ^b^Berná et al. [20]; and ^c^Blazejewski et al. [22].

Phylogenetic relationship of apicomplexans based on WGS data is consistent with the traditional taxonomic classifications (Eucoccidiorida, Haemosporida, and Piroplasmida) of the group, except for *Cryptosporidium* (Figure 1). The latter is now grouped together with gregarines in Cryptogregarinorida in recent taxonomy [23], and both mostly lack apicoplast and mitochondrial genomes. Recent phylogenetic analyses have placed them in two separate clades at the base of Apicomplexa, indicating that they may have different origins [24]. Other apicomplexans have both mitochondrial and apicoplast genomes (Figure 1). However, only a few mitochondrial and apicoplast genomes are well resolved, while *Toxoplasma gondii*, *Neospora caninum*, and other tissue coccidia have many divergent and fragmented copies of the mitochondrial genomes [21].

*Plasmodium* accounts for the most apicomplexan genomic sources (154). Malaria is a major disease causing ~627,000 deaths globally in 2020 [25]. The 154 published *Plasmodium* genomes include 24 reference ones. Among them, four species have over 10 published genomes, including *P. falciparum* (61), *Plasmodium vivax* (17), *Plasmodium vinckei* (10), and *Plasmodium yoelii* (15). In addition to *Plasmodium*, *Babesia* (22) and *Theileria* (16) account for the most genomic sources of Aconoidasida. In contrast to the transmission of *Plasmodium* spp. by mosquitoes, both *Babesia* spp. and *Theileria* spp. are transmitted by ticks, responsible for East Coast fever and tick fever in livestock and humans [26, 27]. Genomes of *Babesia bovis* and *Theileria parva* are ~8 Mb in size, with three chromosomes in *B. bovis* and two chromosomes in *T. parva*.

*Toxoplasma gondii* is a major foodborne pathogen in humans and warm-blood animals. With the availability of good laboratory animal and culture models and advanced genetic tools, *T. gondii* serves as the biological model for apicomplexan research [28]. Currently, 28 *T. gondii* genomes have been published, with the reference genome fully assembled into 14 chromosomes (65.67 Mb). However, recent evidence from Hi–C and TGS analyses suggests that chromosomes VIIb and VIII are, in fact, two fragments of the same chromosome [5, 8]. WGS data are also available for other species of Sarcocystidae, including *N. caninum* (4), *Hammondia hammondi* (1), and *Sarcocystis neurona* (1) (Figure 1) [21, 29].

Most published Eimeriidae genomes are from *Cyclospora* (40) and *Eimeria* (15). *Cyclospora* spp. are food and waterborne parasites that cause diarrhea in humans and animals, with *C. cayetanensis* being the only recognized species in humans and the only *Cyclospora* species for which the whole genome has been sequenced [30]. Although 40 genomes of *C. cayetanensis* have been published, its reference genome of ~44 Mb is highly fragmented with 738 contigs (Figure 1). This is also the case for the genus *Eimeria*. The reference genome of *Eimeria tenella* has 4,665 scaffolds (Figure 1). However, the fragmented genome of *E. tenella* has been updated to the chromosome level (53.25 Mb) using combined NGS, TGS, and Hi–C sequencing [31]. Only 12 reference genomes of *Eimeria* spp. have been published [32]. *Cyclospora* and *Eimeria* genomes cluster together in phylogenetic analysis of WGS, mitochondrial, and apicoplast sequence data, with no clear separation of the two genera [33].

*Cryptosporidium* spp. are important causes of diarrhea and enteric disease in humans and livestock. Nearly 50 *Cryptosporidium* species have been reported [23], but only 16 species have been sequenced for whole genomes (67 assemblies in total). Genomes of this genus have been reported to be ~9 Mb in eight chromosomes (Figure 1). Most WGS data are from *C. parvum* (21) and *C. hominis* (15), the two dominant species in humans. Most of the genome assemblies are fragmented in nature due to the exclusive use of NGS tools. However, several *C. parvum* genomes have been sequenced using combined NGS and TGS technologies and assembled at the chromosome level in the hybrid mode [34, 35, 36].

## 3. Genomic Epidemiology

The accumulation of WGS data makes it possible to use bioinformatics tools in comparative genomic and population genetic characterization of pathogens, improving our understanding of disease epidemiology. Below we show examples of how WGS can help us in understanding the transmission of zoonotic protozoa. Currently, WGS from protozoan parasites have been used effectively in molecular surveillance of diseases and identification of outbreaks (Section 3.1), characterization of the genetic diversity and population structures of pathogens (Section 3.2), tracking the source of infection (Section 3.3), and identification of the genetic determinants of drug resistance (Section 3.4), and other phenotypic traits (Section 3.5).

### 3.1. Molecular Surveillance of Diseases and Identification of Outbreaks

WGS provides comprehensive genomic data for pathogen surveillance. Compared to traditional molecular typing tools, WGS provides sequence data for genetic loci across the entire genome to ensure accurate typing. This allows comprehensive comparison of isolates for genetic diversity, detecting novel mutations at other genetic loci that are often associated with pathogen fitness and biological traits, such as virulence, infectivity, and host preferences. In addition, comparative genomics provides insights into identification of genetic markers for pathogen detection and characterization. This enables advanced typing of isolates without pathogen enrichment and the detection of small outbreaks in defined geographical areas [37], such as the cost-effective SNP-based genotyping method used in investigations of malaria outbreaks in the Greater Mekong Subregion (GMS) [38].

WGS has been used effectively in the investigation of malaria outbreaks. After several rounds of global malaria elimination programs by WHO and some nations, malaria is under control in many countries. However, flare-ups of residual pathogens and reintroduction of new parasites could cause malaria resurgence. WGS monitoring is recommended as it could provide comprehensive molecular surveillance data to guide malaria control [39]. For example, Cape Verde is now in the pre-elimination phase of malaria control, as only a few cases have been reported each year except for an outbreak in 2017. However, results of WGS analysis indicate that clonal expansion of local parasites has occurred and currently most parasites carry drug resistance-associated mutations [40]. Similarly, reduced genetic diversity was reported in a WGS-based analysis of *P. falciparum* samples from an outbreak in Laos, suggesting a recent selective sweep [41]. As some other countries such as Thailand is in the pre-elimination phase, WGS-based molecular surveillance is increasingly used in the identification of the transmission characteristics of residual parasites [42]. In some cases, the analysis of mitochondrial genomes is used in molecular surveillance. For example, its use showed that *P. simium*, which is genetically related to *P. vivax*, was responsible for 2015 and 2016 outbreaks of malaria in the Atlantic Forest of southeastern Rio de Janeiro, Brazil [43].

**Figure 2:**
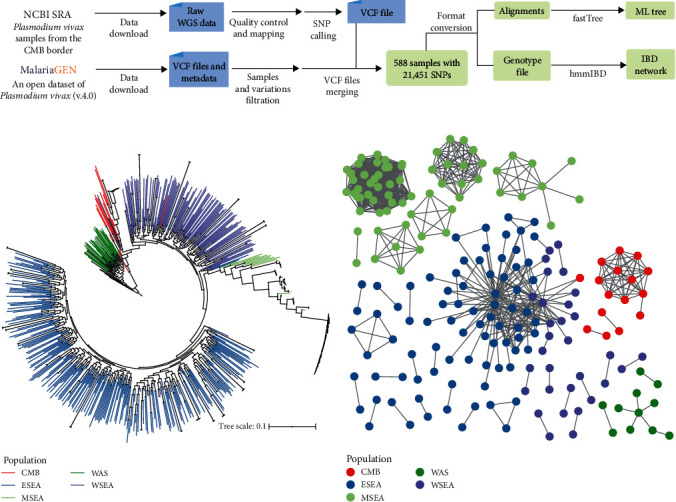
*Plasmodium vivax* outbreak investigation on the China–Myanmar border (CMB) by analysis of whole genome sequencing (WGS) data. (a) Schematic illustration of analysis of *P. vivax* WGS data for outbreak detection. Briefly, raw WGS data from CMB samples were downloaded from NCBI for the identification of SNPs. Whole-gneome variations in samples from other Asian countries were obtained from MalariaGEN (https://www.malariagen.net/). The variants are stored in a standardized textual Variant Call Format (VCF) file. The two SNP datasets were then merged. Biallelic SNPs with Phred quality score (QUAL) and mapping depth greater than 30, read depth greater than 3, and missing rate less than 5% were used for further analysis. (b) Maximum likelihood (ML) tree of *P. vivax*. SNPs were concatenated into alignments for tree building using FastTreeMP v2.11.1 [47]. (c) Identity-by-descent (IBD) network of *P. vivax*. The VCF file above was converted into a genotype matrix, and IBD was calculated using hmmIBD v2.0.4 [48]. Each node in the network represents a sample, and an edge is drawn between two genomes that share more than 90% of IBD. Branches (b) or shapes (c) in different colors correspond to sample sources, including CMB, Eastern Southeast Asia (ESEA), the Maritime Southeast Asia (MSEA), Western Asia (WSA), and Western Southeast Asia (WSEA). Based on data and analytical approaches of Brashear et al. [44] and the *P. vivax* Genome Variation Project (Pv4 dataset) [45].

Here we showcase the utility of WGS data of *P. vivax* isolates in the investigation of outbreaks along the China–Myanmar border (CMB) by reanalyzing data mostly from a previous study using the same approach [44, 45]. For most of the GMS, malaria cases have decreased each year. However, the incidence of *P. vivax* malaria is increasing on the CMB [46]. In a recent study, WGS analysis was used to compare the *P. vivax* population from the CMB with populations in neighboring areas [44]. Using a similar bioinformatic pipeline (Figure 2(a)), a reanalysis of the data together with additional MalariaGEN data from Asia [45] has supported the genetic uniqueness of *P. vivax* from CMB. Most CMB genomes are clustered in one unique clade in a maximum likelihood analysis of the SNP matrix generated from read mapping (Figure 2(b)). The identity-by-descent (IBD) analysis of the SNPs has also shown the formation of a distinct lineage of the same origin (Figure 2(c)). The analysis suggests an independent population of *P. vivax* of the same ancestral origin in recent malaria outbreaks at CMB. This finding is valuable in the formulation of malaria elimination strategy for this area.

The use of WGS for molecular surveillance and outbreak identification in toxoplasmosis has been hampered by the limited availability of *T. gondii* DNA from clinical samples, a challenge also faced by other parasites [49]. There is currently no standard molecular tool for *T. gondii* genotyping, making it difficult to compare or integrate surveillance data [50]. Furthermore, the limited sequence information available cannot distinguish atypical or recombinant strains. A recently developed method, circular nucleic acid enrichment reagent synthesis (CNERs), has been used to generate whole-genome enrichment (WGE) probes [51]. This method has also been successfully applied to enrich *T. gondii* DNA from various sources. The WGE-CNERs method for *T. gondii* can detect as few as 50 oocysts per ml of oyster hemolymph, which is promising for genomic surveillance of *T. gondii* from food, environmental, and clinical samples.

In addition, WGS has also been used to investigate cryptosporidiosis outbreaks, such as the emergence of a *C. hominis* subtype in recent cryptosporidiosis outbreaks [52]. In this study, diverse population and evolutionary genetic tools were used to trace the sources of a hypertransmissible subtype responsible for recent cryptosporidiosis outbreaks in the United States. The subtype was shown to have three major variants, initially derived from East Africa and Europe but have gone through secondary recombination with local isolates and each other, leading to the appearance of multiple genomic variants of the same *gp60* subtype. Subsequent selection in mucin glycoprotein genes, as evident in the presence of selective sweep at these genetic loci, has led to the emergence of a dominant variant in sporadic cases and outbreaks [52].

### 3.2. Characterization of Genetic Diversity and Population Structures of Pathogens

Genomic differences between isolates provide fingerprints that reveal population structure and evolutionary history. At the species level, genomic analyses frequently focus on the identification of genetic differences that help understand the evolution of organisms. Comparative genomics allows us to identify similarity in genome organizations [20] and build more authentic phylogenetic trees [53]. For example, results of comparative genomic analyses suggest that the apicomplexans have evolved from free-living photosynthetic organisms [24]. Among apicomplexan parasites, reductive evolution of mitosome metabolism and subtelomeric genes has been observed within the *Cryptosporidium* lineage, leading to reduced host range and pathogenicity in some species distant from *C. parvum* [54, 55].

Within the species of public health and veterinary importance, as the sequencing cost decreases, more isolates of different phenotypes are being sequenced. Unique genomic variations can be identified in the WGS data, including SNPs and small INDELs, gene gains and losses, selective pressure, and genetic recombination [56]. In particular, genetic recombination speeds up the evolutionary process, leading to the emergence of new genotypes of different phenotypic traits. This brings additional challenges to parasite elimination.

**Figure 3:**
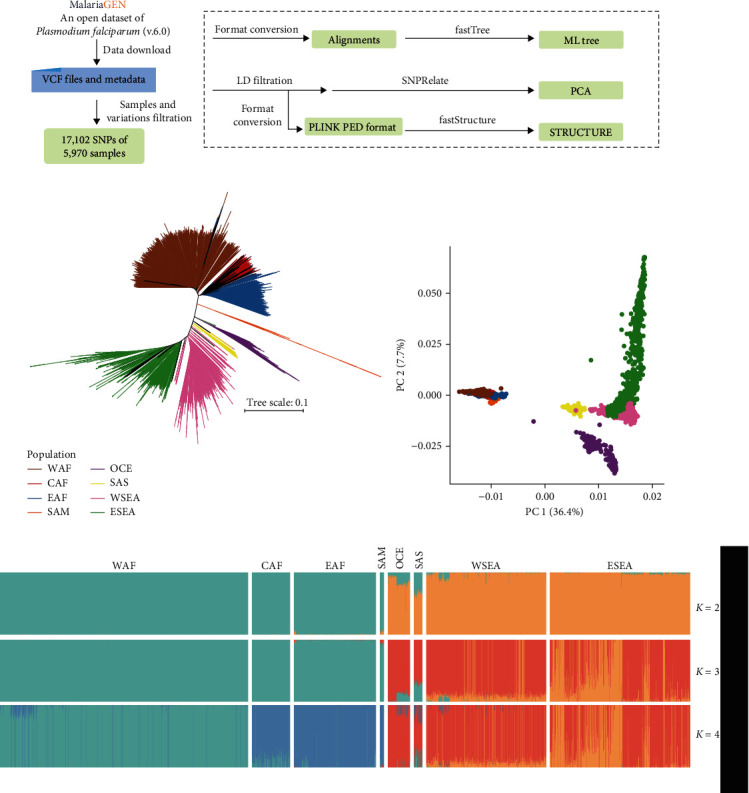
Population structure analysis of whole genome sequencing (WGS) data from *Plasmodium falciparum*. (a) Schematic illustration of population structure analysis of WGS data from *P. falciparum*. Whole-gneome variations of representative samples were obtained from *P. falciparum* Community Project (Pf6) of MalariaGEN (https://www.malariagen.net/). They were filtered according to the quality control annotated in the metadata file and README statement. In addition, biallelic SNPs in coding regions with QUAL and mapping depth greater than 30, depth greater than 3, and missing rate less than 5% were used for further analysis. (b) Maximum likelihood tree of *P. falciparum*. SNPs were concatenated into alignments for tree construction using FastTreeMP v2.11.1 [47]. Samples were colored according to genographic regions, including West Africa (WAF), Central Africa (CAF), East Africa (EAF), South America (SAM), Oceania (OCE), South Asia (SAS), West Southeast Asia (WSEA), and East Southeast Asia (ESEA). (c) Principal component analysis (PCA) of 14,063 unlinked SNPs. Each dot represents a strain and the color corresponds to (b). The PCA analysis was performed with SNPRelate [105]. (d) Population sturcture of *P. falciparum* revealed by analysis of the SNP data with fastStructure [106] at *K* values of 2–4. The proportion of colored regions in each bar indicates the corresponding ancestral components. Based on data and analytical approaches of the published *P. falciparum Community Pr*oject (Pf6) [58].

Genetic differences between or within populations may provide insights into pathogen evolution. In many apicomplexans such as *P. falciparum* and *P. vivax*, isolation-by-distance plays an important role in shaping their population structures [45, 57]. The increasing numbers of *Plasmodium* genomes have greatly facilitated studies of the evolution of the pathogens and molecular epidemiology of malaria. In recent years, the global data-sharing network Malaria Genomic Epidemiology Network (MalariaGEN) has curated genomic variation data on 20,864 *P. falciparum* samples from 82 partner studies in 33 malaria-endemic countries [57]. Here, we reanalyzed part of the WGS data using the same approach [58] to present the *P. falciparum* population structure in a similar way (Figure 3(a)). Results of phylogenetic, principal component, and population structure analyses have identified the presence of geographic segregation in *P. falciparum* populations, with isolates from different regions of the world forming their own clusters (Figures 3(b), 3(c), and 3(d)). Such large-scale data have provided access to a comprehensive study of the global diversity of *P. falciparum*. However, the broad scale of genetic differences could conceal subpopulations in local areas, such as multiple populations circulating at the Thai–Myanmar border [59] and in sub-Saharan Africa [60]. Nonetheless, the accumulation of WGS data around the world makes it possible to investigate the evolution and migration of *P. falciparum* at both local and global levels for different purposes.

Similarly, results from WGS analysis of 62 globally distributed *T. gondii* isolates support the existence of geographic segregation of population structure, and the inheritance of large haploblocks shared between related strains has also been identified, suggesting that recombination also accelerates the evolutionary adaptation of *T. gondii* [61]. TGS-based comparative genomic analysis of *T. gondii* has also revealed changes in copy number and order of tandem gene families due to sexual recombination [5]. Additional analysis of newly sequenced genomes has also supported the existence of hybrid *T. gondii* genomes, with evidence of positive selection acting on a unique haplotype (~100 kb) on chromosome 1a [62]. It has been suggested that the emergence and transmission of this haplotype may have accompanied the domestication of cats from the Old World to the New World.

**Figure 4:**
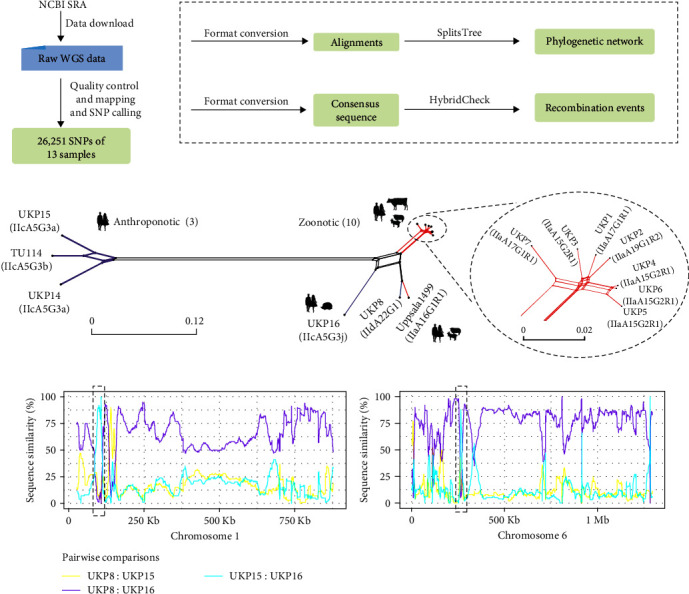
Detection of recombination events among different *C. parvum* subtypes. (a) Schematic illustration of WGS analysis to detect recombination events in *C. parvum* using a published dataset. Raw WGS data were downloaded from NCBI and SNPs were identified as described in Figure 2. (b) Neighbor-joining phylogenetic network was constructed with SplitsTree v4 [107]. Branches were colored according to the *gp60* subtype family, including the anthroponotic IIc and the zoonotic IIa and IId subtype families. (c) Pairwise sequence similarity between three *C. parvum* genomes. Analysis of recombination event of the possible progeny UKP16 and two potential parents UKP15 and UKP8 were performed using HybridCheck [108]. Two recombination events located on chromosomes 1 and 6 are depicted with dashed black frames. Based on data and analytical approaches of Nader et al. [53] and Troell et al. [66].

As indicated above, population genetic analyses have shown that recombination between different *C. parvum* subtypes can lead to the emergence of new populations [63, 64]. Genetic recombination and selective sweeps have resulted in the formation of more adapted populations of *C. hominis*, which are now the dominant subtypes for outbreaks in high-income countries [52, 65]. For example, a recent study of *Cryptosporidium* has revealed genetic exchanges between anthroponotic and zoonotic *C. parvum*, leading to the emergence of novel subtypes [53]. Here, we showcase the utility of WGS of *C. parvum* isolates in the identification of recombination events using a pipeline similar to the one in the publication (Figure 4(a)) [53, 63]. Results of phylogenetic network and recombination event analyses of 26,251 SNPs among 13 published *C. parvum* genomes [53, 66] indicate possible occurrence of recombination events in isolate UKP16 of the IIcA5G3j subtype (Figure 4(b)). The recombination leads to the presence of a mosaic sequences across the entire genome, with UKP8 (IIdA22G1 subtype) and UKP15 (IIcA5G3a subtype) being the likely parents (Figure 4(c)). As a result, the IIcA5G3j subtype has expanded its host range from the anthroponotic IIcA5G3a subtype due to sequence introgression from the zoonotic IIdA22G1 subtype. This serves as a good example for the utility of WGS data in improved understanding of evolutionary history and phenotypic diversity of pathogens. This is also the case in *C. parvum* and *C. hominis* [53, 63, 64]. For the anthroponotic *C. hominis* commonly found in resource-limited countries, genomes form country-specific clusters in phylogenetic analysis of WGS data. However, one virulent subtype commonly found in some resource-limited countries, IbA10G2, has become the dominant *C. hominis* in industrialized nations due to frequent international travels. It has a conserved genome divergent from other *C. hominis* subtypes, a feature underlying its high rates of direct human-to-human transmission [65].

Other statistics can be used in measurements of genetic diversity of parasite populations based on the frequency of alleles, such as nucleotide diversity (*Pi*) and fixation index (*F*_st_) [67]. WGS data also enable genome-wide scanning for genes under selection during the evolutionary history of parasites through genome-wide association studies (GWAS) [68], cross-population extended haplotype homozygosity (XP-EHH) test, and standardized integrated haplotype score (|iHS|) [69]. The recent explosion of WGS data and information on identified drug resistance genes allows the implementation of a deep learning approach (convolutional neural network, CNN) in the analysis of *P. falciparum* and *P. vivax* genomes for the identification of recent positive selection [70].

### 3.3. Molecular Tracing of Infection Sources and Dispersal of Pathogens in Endemic Areas

Tracking the infection source is an important part of epidemiological investigations and is essential to disease control and prevention. This is complicated by increased international travels in recent years, which promotes transterritory and intercontinental transmission of pathogens [52]. In addition, concurrent infections of multiple genotypes or subtypes of the same pathogen are common in endemic areas [71]. As a result, gene flow can occur between two or more genetically divergent populations, generating variants with novel genetic and phenotypic traits [63]. These make it more difficult to trace the source of pathogens.

WGS data and population genomics are valuable for characterizing infection sources and transmission dynamics of pathogens [60, 72]. Patterns of spatiotemporal pathogen dispersal are difficult to identify with conventional surveillance tools but could be assessed by comparative analyses of WGS data for genetic diversity, pairwise differentiation, ancestral relationships, and genomic regions or loci associated with geographic segregation or under selection [60]. With more abundant WGS data worldwide, population structures and gene flow events within species can help elucidate the origin and dispersal of pathogens, changes in fitness and phenotypes, and mechanisms for the emergence of novel genotypes or subtypes [39].

For example, recent population genomic analyses of *P. falciparum* suggest that American populations originated from Africa via two introductions but have experienced adaptive evolution to new human populations and mosquito species since their introduction from Africa [73]. IBD analysis of WGS data from *P. falciparum* isolates collected from two distant port cities on the Colombia–Pacific coast revealed unexpected connectivity through marine traffic, making the formulation of targeted intervention measures possible [74]. IBD analysis of polygenomic infections revealed cotransmission of genetically related *P. falciparum* parasites in Thiès, Senegal [75]. In addition, the extent of multiplicity of infection (MOI) can be estimated from raw WGS data [67].

Similar approaches have been used to track infection and spread of other *Plasmodium* species. Recent introgression events between two zoonotic *Plasmodium knowlesi* subpopulations, which have different macaque reservoir hosts and separate sympatric transmission cycles, have altered the potential for *P. knowlesi* transmission by mosquito vectors in Malaysian Borneo [76]. In the aforementioned study, genetic similarity between African and South Asian populations suggested that *P. vivax* may have been reintroduced into Africa from South Asia [77]. Indeed, population genomic analysis of 447 sets of WGS data from *P. vivax* isolates from 21 different countries and 19 *P. vivax*-like isolates from great apes suggests that *P. vivax* may have a single origin in South Asia [78]. In the analysis, intrapopulation genetic diversity decreased with increasing distance from Southeast Asia, suggesting a founder effect in evolutionary history. Recent population genomic evidence also suggests a unique evolutionary history of *P. simium*, which may have evolved from New World *P. vivax* lineages and been transmitted from humans to nonhuman primates [79]. In addition, machine learning approaches incorporating hierarchical fixation index and decision tree analyses of WGS data have been used effectively to identify imported *P. vivax* malaria [80].

**Figure 5:**
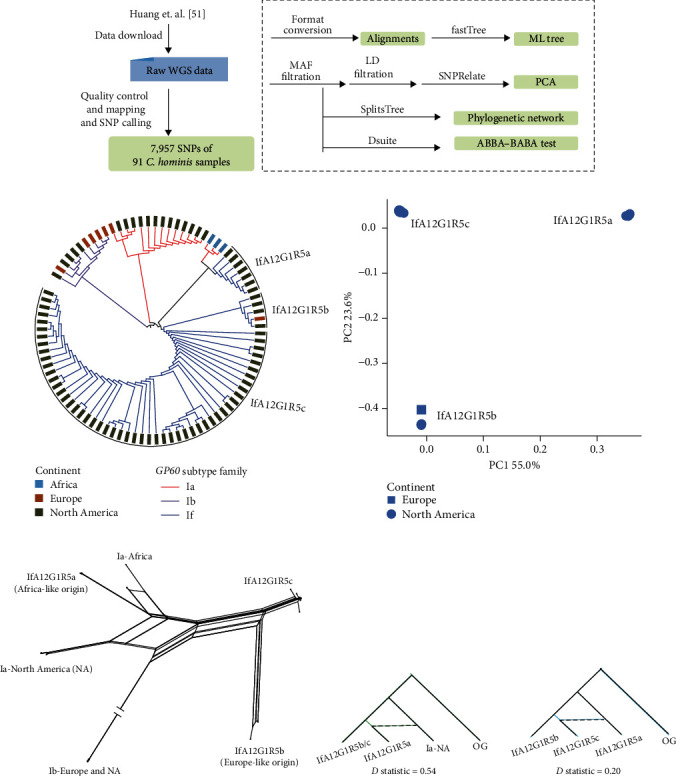
Origin and dispersal of an emerging *C. hominis* subtype. (a) Schematic illustration of the WGS analysis to investigate the origin and dispersal of a novel hypertransmissible *C. hominis* subtype (IfA12G1R5). Raw WGS data of 91 *C. hominis* samples were downloaded from NCBI. Reads were processed and whole genome variations were identified as described by Huang et al. [52]. (b) Maximum likelihood tree of *C. hominis*. SNPs were concatenated into alignments for tree building using FastTreeMP v2.11.1 [47]. The color of each bar corresponds to the source of each genome and the color of each branch corresponds to the *gp60* subtype family of each genome. (c) Principle component analysis (PCA) of 1,088 unlinked SNPs from the IfA12G1R5 subtype. Squares represent samples collected from Europe and dots represent genomes from North America. The PCA analysis was performed with SNPRelate [105]. (d) Phylogenetic network of *C. hominis* based on analysis of concatenated SNPs. (e) Introgression events between different populations. With the assumed phylogenetic relationship (((P1, P2), P3), OG), *D* statistics were used to assess the introgression between P2 and P3. A *D* value greater than 0 indicates the presence of sequence introgression. The *D* statistics were calculated using Dsuite [109]. Based on data and analytical approaches of Huang et al. [52].

Here, we showcase the utility of WGS analysis in the investigation of the origin and spread of the hypertransmissible *C. hominis* subtype IfA12G1R5 in the USA, as mentioned above [52]. We selected 91 genomes, including all published IfA12G1R5 genomes, and analyzed the data using a similar bioinformatic pipeline (Figure 5(a)). The phylogenetic tree constructed with 7,957 SNPs has revealed that this subtype is divided into three subpopulations with little intrasubpopulation diversity, suggesting the recent emergence of them (Figure 5(b)). This is supported by the principle component analysis (PCA) (Figure 5(c)).

Notably, the IfA12G1R5a group from the USA was closely related to the Ia subtype family in Africa, suggesting a potential African origin for this group. Gene flow is also apparent between them (Figure 5(d)). For the IfA12G1R5b group, one genome obtained from Sweden in 2013 clusters with several genomes obtained from the USA during 2016–2017. The high genomic similarity between them suggests a potential European origin for the IfA12G1R5b group. In addition, the aforementioned second genetic recombination events are present between these three IfA12G1R5 groups (Figure 5(d)), which is supported by the result of the ABBA-BABA test (Figure 5(e)).

### 3.4. Identification of Genetic Determinants of Drug Resistance

The administration of drugs to control parasitic diseases eliminates the susceptible populations of parasites after the treatment. This selective pressure on the parasites creates a population bottleneck, resulting in the increased frequency of the drug-resistant alleles at relevant genetic loci and the linked genetic loci on the same haplotype background (selective sweep) [81]. The emergence of drug resistance has been a major problem affecting the control and prevention of parasitic diseases, especially in the eradication of malaria and other protozoa diseases of significant public health importance [82].

With the rapid accumulation of WGS data, it is now feasible to identify new biomarkers of drug resistance by assessing the association between genotypes and phenotypes of parasites using bioinformatic approaches. For example, despite the *pfcrt* K76T is a widely used molecular marker for chloroquine (CQ) resistance in *P. falciparum* [83], GWAS on CQ-resistant (17), and CQ-sensitive parasites (18) containing 34,196 whole genome SNPs (wgSNPs) in French Guiana identified the *pfcrt* C350R mutation associated with CQ resistance (CQR) [84]. More recently, population genetic analyses on 321 isolates from Gambia identified another mutation (*pfaat1* S258L) on chromosome 6 associated with CQR [85]. Several LD-based methods, site frequency spectrum statistics, and machine learning approaches can also be used to investigate putative selective sweeps in each population to determine genetic bases of drug resistance [70, 81].

Comparative analysis of WGS data allows us to associate genetic variations with drug resistance phenotype, facilitating the development of biomarkers for molecular surveillance of drug resistance [67]. Known drug resistance-associated genes have been used in tracking the occurrence and dispersal of drug resistance in disease-endemic areas. Genomes of unknown phenotypes can be examined for drug resistance-associated genes by mapping genomic variations to known drug-resistant loci [40, 41]. Recently, markers have been developed to provide information on drug resistance in *P. falciparum* samples from patients with high parasitemia, including *pfdhfr*, *pfdhps*, *pfk13*, *pfmdr1*, *pfcrt*, and *pfama1* [40]. The open datasets of *P. falciparum* and *P. vivax* also provide the distribution of drug resistance genotypes based on the known markers of drug resistance [45, 57].

**Figure 6:**
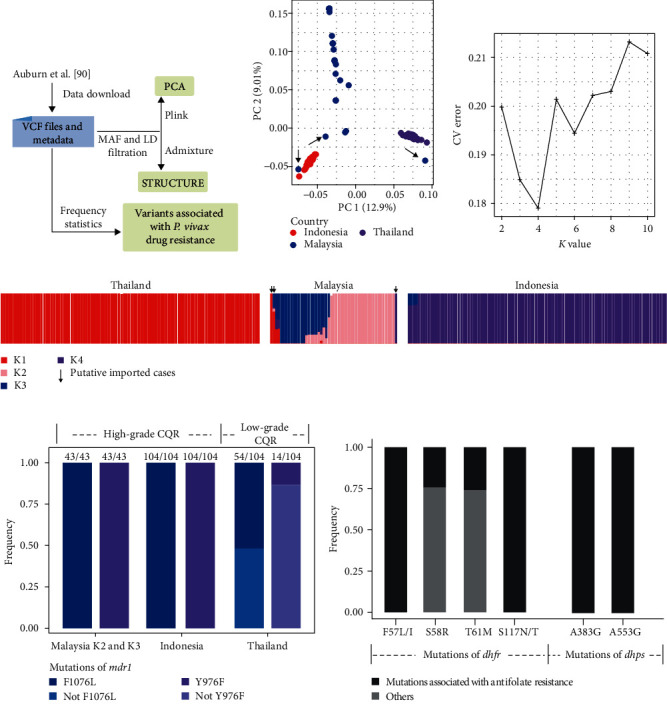
Identification of possible occurrence of drug resistance in Plasmodium *vivax* in Malaysia. (a) Schematic illustration of WGS analysis for the identification of potential drug resistance in a pre-elimination *P. vivax* population in Malaysia. Whole-gneome variations from 259 samples were obtained from MalariaGEN (https://www.malariagen.net/) and the VCF file was used in the following analyses. (b) Principal component analysis of *P. vivax*. Each node represents one genome and is colored according to its source. The analysis was performed with plink v1.9. (c) Cross-validation results of *K* values of 2–10 using Admixture v1.3 [110]. The cross-validation error is lowest at *K* = 4. (d) Population structure of *P. vivax* at *K* = 4 based on the analysis of the data using Admixture. (e) The frequency of variations potentially associated with *P. vivax* chloroquine resistance (CQR) in three countries with different grades of CQR. (f) Frequency of other variations associated with *P. vivax* resistance to antifolate in Malaysia. Based on data and analytical approaches of Auburn et al. [86].

Here, we showcase the utility of WGS analysis in identifying possible occurrence of drug resistance in a pre-elimination *P. vivax* population in Malaysia [86]. WgSNPs of 259 samples were obtained from MalariaGEN. Population structure and allele frequencies were then analyzed (Figure 6(a)). Results of the PCA analysis indicate the presence of three parasite populations associated with sample sources (Figure 6(b)). STUCTURE analysis also shows a significant genomic identity between three Malaysian genomes and those from Indonesia and Thailand, indicating the presence of possible imported infections (Figures 6(c) and 6(d)). However, most of the Malaysia genomes display greater divergence from those obtained from Indonesia and Thailand, and further cluster into two groups (K2 and K3). The 26 K2 genomes have higher genetic identity, with median differences of only five SNPs [86]. At the *pvmdr1* (PVP01_1010900) locus, they have Y976F and F1076L mutations, which have been associated with chloroquine resistance (CQR), with the former also linked to resistance to amodiaquine (AQ) and sulfadoxine-pyrimethamine (SP) [87]. Among the genomes analyzed, all genomes in Indonesia (104/104, 100%) and K2 and K3 genomes in Malaysia (43/43, 100%) have these mutations, which is consistent with the high-grade CQR present in these areas (Figure 6(e)). Similarly, these genomes have the *pvdhfr* (PVP01_0526600) S58R, T61M, and S117N/T mutations and the *pvdhps* (PVP01_1429500) A383G and A553G mutations (Figure 6(f)), which are molecular markers for antifolate (AQ and SP) resistance [87]. If confirmed, these data on the possible prevalence of CQ, AQ, and SP resistance are very useful in formulating *P. vivax* elimination strategies. They may provide insight into the emergence of an adaptive *P. vivax* population in Malaysia. However, CQR in *P. vivax* is a complicated issue, and we should be cautious about the association of pvmdr1 mutations with CQR. The in *vivo* resistant phenotype of *P. vivax* infection is variable, and there is no *in vitro* CQR assay for this species. Furthermore, upregulation of the *pvcrt* gene has been shown to be a mechanism of drug resistance in *P. vivax* [88].

### 3.5. Identification of Genetic Determinants for Other Phenotypic Traits

Comparative genomics can be used effectively in the identification of genetic mechanisms associated with host preference. For example, *T. gondii* and *N. caninum* are two cyst-forming coccidian parasites with distinct host preferences [20]. *T. gondii* infects a wide range of warm-blooded vertebrates including humans, but uses only felids as the definitive hosts. In contrast, *N. caninum* infects mostly herbivores, has never been reported in humans, and uses canines as the definitive hosts. Comparative genomic analyses have identified a large number of conserved genes, as well as some species-specific gene families involved in host-parasite interactions [61]. For example, the *ROP18* has been reported to be associated with *T. gondii* virulence in mice [89], but appeared to be a pseudogene in *N. caninum*, which may explain its inability to phosphorylate host immunity-related GTPases [90]. Within *C. parvum*, results of comparative genomics suggest that the gains and losses of subtelomeric genes encoding several secretory protein families could be determinants of differences in host preference among different *gp60* subtype families [63, 91]. Similarly, Duffy binding protein 1 amplification has been associated with the adaptation by *P. vivax* to Duffy-negative populations in Ethiopia [92].

**Figure 7:**
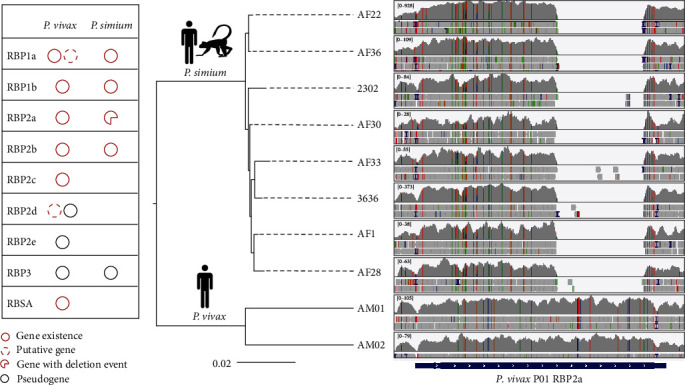
Identification of genes associated with host preference in *Plasmodium simium* by comparative genomic analysis. (a) Comparison of the reticulocyte-binding protein (RBP) family between *P. simium* and *Plasmodium vivax*. Each circle represents the existance of RBPs. Dashed and black circles represent putative gene and pseudogene, repectively. A broken circle represents gene with a deletion event. (b) Read mapping results of RBP2a. The tree on the left was constructed using whole genome SNPs from eight *P. simium* samples and two *P. vivax* samples. The cartoons at each node indicate the host of parasites in the clade. Read mapping results are viewed with IGV (https://www.igv.org/). The analysis was based mainly on data and analytical approaches of Mourier et al. [93].

Here, we showcase the utility of WGS analysis in investigating the host switching of *Plasmodium simium* [93]. Formerly known as a nonhuman primate malaria species, *P. simium* has recently been found to infect humans [43]. Comparative genomics analysis between *P. simium* and *P. vivax* revealed a reduced number of proteins associated with malaria infection in the former (Figure 7). Among them, the reticulocyte-binding proteins (RBPs) are important for host cell invasion. The *P. vivax* P01 genome contains 11 RBP genes, whereas *P. simium* harbors only five such genes (Figure 7(a)). Furthermore, a large deletion was observed in RBP2a, which might affect host cell recognition by *P. simium* (Figure 7(b)). Compared with *P. vivax*, homology modeling indicates that the large deletion in *P. simium* likely affects the disordered regions of RBP2a, which may play a role in binding unknown ligands, leading to its infectivity in monkeys [93].

The expansion of polymorphic gene families, especially those encoding secretory proteins, is a common event along with changes in host preference or virulence. By comparing the average sequence read depth across the genomes of 62 globally distributed *T. gondii* isolates, genes with CNV were identified, many of which encode secretory or surface proteins considered as secretory pathogenesis determinants (SPDs) [61]. Gene expansion events have also been detected in other parasites, such as the *var* (variant antigen) genes in *P. falciparum* [14, 94], *vir* (part of the *Plasmodium* interspersed repeats superfamily) genes in *P. vivax* [95], and *vsg* (encoding variant surface glycoprotein) genes in *Trypanosoma brucei* [96]. Further evidence for genetic determinants of phenotypic traits will emerge as more WGS data are generated from parasites with different phenotypic traits, providing new strategies for the development of disease control and treatment measures.

## 4. Concluding Remarks

WGS has modernized the infectious disease epidemiology, bringing it to the era of precision public health [1]. However, among the thousands of species of protozoan parasites, only a small number with major veterinary and public health importance have been sequenced for whole genomes. More WGS data are needed for protozoan parasites to promote the understanding of their biology and transmission and the development of public health surveillance systems.

Comparative genomics has played a major role in revealing hidden genetic diversity that is difficult to detect using traditional molecular diagnostic tools, providing the basis for rapid detection of outbreaks and transmission clusters, accurate tracking of infection sources, and the development of advanced molecular surveillance systems [9]. The decreasing sequencing costs of NGS has promoted the use of WGS data in other advanced characterization of pathogens, such mechanisms involved in pathogen evolution and emergence of new subtypes and variants, genetic determinants of various phenotypic traits such as drug resistance, host adaptation, and transmissibility and virulence. The use of population and evolutionary genetic analyses of large-scale WGS data in recent years has led to better understand pathogen origins, transmission patterns, evolutionary histories, and selection pressure.

Currently, many protozoan parasites lack WGS data to support molecular epidemiological studies. What we know for now is just the tip of the iceberg. The application of WGS for molecular tracing of infection sources and the spread of some pathogens is hampered by many other factors, such as the lack of key metadata for historical samples, the lack of communications and collaboration in the community, and the multiplexity infection [60]. Moreover, the limited length of sequencing reads prevents us from fully assessing the role of copy number variations of genes and structural variations of genomes in the transmission of protozoa pathogens [97]. Recently, the application of TGS has greatly improved the quality of reference genomes, allowing us to locate more complex structural variations in *P. knowlesi* [98], *B. bovis* [99], *C. parvum* [34], *T. gondii*, and *N. caninum* [5]. These well-assembled genomes facilitate the characterization of host adaptation, detection of virulence determinants and novel drug targets, and advanced studies of pathogen genetics and evolution. However, many reference genomes for pathogens are still fragmented. These physical gaps and undistinguished bases might contain important information.

Some novel WGS analyses may greatly facilitate genomic epidemiological studies of parasitic protozoa that lack effective isolation methods. Direct WGS of samples from the host and environment can enable a much faster response to outbreaks [100, 101]. Metagenomics allows the detection of multiple parasites in samples simultaneously, providing us a new revenue of comprehensive surveillance of infectious diseases [102]. Recently, various artificial intelligence tools have been introduced into WGS data analysis [80, 103, 104]. These new developments will likely promote the use of genomic epidemiology in the investigations and surveillance of diseases caused by parasitic protozoa.

## Data Availability

All data supporting the results of this review were obtained from published studies as described in the main text. The procedures used to process and analyze the data are described in the legend for each figure.

## References

[1] Armstrong G. L., MacCannell D. R., Taylor J. (2019). Pathogen genomics in public health. *The New England Journal of Medicine*.

[2] Dartois V. A., Rubin E. J. (2022). Anti-tuberculosis treatment strategies and drug development: challenges and priorities. *Nature Reviews Microbiology*.

[3] Dallman T. J., Jalava K., Verlander N. Q. (2022). Identification of domestic reservoirs and common exposures in an emerging lineage of Shiga toxin-producing *Escherichia coli* O157: H7 in England: a genomic epidemiological analysis. *Lancet Microbe*.

[4] Wu F., Zhao S., Yu B. (2020). A new coronavirus associated with human respiratory disease in China. *Nature*.

[5] Xia J., Venkat A., Bainbridge R. E. (2021). Third-generation sequencing revises the molecular karyotype for *Toxoplasma gondii* and identifies emerging copy number variants in sexual recombinants. *Genome Research*.

[6] Munnink B. B. O., Nieuwenhuijse D. F., Stein M. (2020). Rapid SARS-CoV-2 whole-genome sequencing and analysis for informed public health decision-making in the Netherlands. *Nature Medicine*.

[7] Lieberman-Aiden E., van Berkum N. L., Williams L. (2009). Comprehensive mapping of long-range interactions reveals folding principles of the human genome. *Science*.

[8] Bunnik E. M., Venkat A., Shao J. (2019). Comparative 3D genome organization in apicomplexan parasites. *Proceedings of the National Academy of Sciences of the United States of America*.

[9] Gardy J. L., Loman N. J. (2018). Towards a genomics-informed, real-time, global pathogen surveillance system. *Nature Reviews Genetics*.

[10] Pisarski K. (2019). The global burden of disease of zoonotic parasitic diseases: top 5 contenders for priority consideration. *Tropical Medicine and Infectious Disease*.

[11] Cowell A. N., Winzeler E. A. (2019). Advances in omics-based methods to identify novel targets for malaria and other parasitic protozoan infections. *Genome Medicine*.

[12] Barrow P., Dujardin J. C., Fasel N. (2020). Viruses of protozoan parasites and viral therapy: is the time now right?. *Virology Journal*.

[13] Domagalska M. A., Dujardin J.-C. (2020). Next-generation molecular surveillance of TriTryp diseases. *Trends in Parasitology*.

[14] Gardner M. J., Hall N., Fung E. (2002). Genome sequence of the human malaria parasite *Plasmodium falciparum*. *Nature*.

[15] Xu P., Widmer G., Wang Y. (2004). The genome of *Cryptosporidium hominis*. *Nature*.

[16] Abrahamsen M. S., Templeton T. J., Enomoto S. (2004). Complete genome sequence of the apicomplexan, *Cryptosporidium parvum*. *Science*.

[17] Gajria B., Bahl A., Brestelli J. (2008). ToxoDB: an integrated *Toxoplasma gondii* database resource. *Nucleic Acids Research*.

[18] Emms D. M., Kelly S. (2019). OrthoFinder: phylogenetic orthology inference for comparative genomics. *Genome Biology*.

[19] Minh B. Q., Schmidt H. A., Chernomor O. (2020). IQ-TREE 2: new models and efficient methods for phylogenetic inference in the genomic era. *Molecular Biology and Evolution*.

[20] Berná L., Marquez P., Cabrera A., Greif G., Francia M. E., Robello C. (2021). Reevaluation of the *Toxoplasma gondii* and *Neospora caninum* genomes reveals misassembly, karyotype differences, and chromosomal rearrangements. *Genome Research*.

[21] Namasivayam S., Baptista R. P., Xiao W. (2021). A novel fragmented mitochondrial genome in the protist pathogen *Toxoplasma gondii* and related tissue coccidia. *Genome Research*.

[22] Blazejewski T., Nursimulu N., Pszenny V. (2015). Systems-based analysis of the *Sarcocystis neurona* genome identifies pathways that contribute to a heteroxenous life cycle. *ASM Journal*.

[23] Ryan U. M., Feng Y., Fayer R., Xiao L. (2021). Taxonomy and molecular epidemiology of *Cryptosporidium* and *Giardia—*a 50 year perspective (1971–2021). *International Journal for Parasitology*.

[24] Mathur V., Kolísko M., Hehenberger E. (2019). Multiple independent origins of apicomplexan-like parasites. *Current Biology*.

[25] World Health Organization (2021). World malaria report 2021. https://www.who.int/teams/global-malaria-programme/reports/world-malaria-report-2021.

[26] Brayton K. A., Lau A. O. T., Herndon D. R. (2007). Genome sequence of *Babesia bovis* and comparative analysis of apicomplexan hemoprotozoa. *PLoS Pathogens*.

[27] Gardner M. J., Bishop R., Shah T. (2005). Genome sequence of *Theileria parva*, a bovine pathogen that transforms lymphocytes. *Science*.

[28] Khan A., Taylor S., Su C. (2005). Composite genome map and recombination parameters derived from three archetypal lineages of *Toxoplasma gondii*. *Nucleic Acids Research*.

[29] Walzer K. A., Adomako-Ankomah Y., Dam R. A. (2013). *Hammondia hammondi*, an avirulent relative of *Toxoplasma gondii*, has functional orthologs of known *T. gondii* virulence genes. *Proceedings of the National Academy of Sciences of the United States of America*.

[30] Herwaldt B. L. (2000). *Cyclospora cayetanensis*: a review, focusing on the outbreaks of cyclosporiasis in the 1990s. *Clinical Infectious Diseases*.

[31] Aunin E., Böhme U., Blake D. (2021). The complete genome sequence of *Eimeria tenella* (Tyzzer 1929), a common gut parasite of chickens. *Wellcome Open Research*.

[32] Blake D. P. (2015). *Eimeria* genomics: where are we now and where are we going?. *Veterinary Parasitology*.

[33] Liu S., Wang L., Zheng H. (2016). Comparative genomics reveals *Cyclospora cayetanensis* possesses coccidia-like metabolism and invasion components but unique surface antigens. *BMC Genomics*.

[34] Baptista R. P., Li Y., Sateriale A. (2022). Long-read assembly and comparative evidence-based reanalysis of *Cryptosporidium* genome sequences reveal expanded transporter repertoire and duplication of entire chromosome ends including subtelomeric regions. *Genome Research*.

[35] Menon V. K., Okhuysen P. C., Chappell C. L. (2022). Fully resolved assembly of *Cryptosporidium parvum*. *Gigascience*.

[36] Huang W., Tang K., Chen C. (2024). Sequence introgression from exogenous lineages underlies genomic and biological differences among *Cryptosporidium parvum* IOWA lines. *Water Research*.

[37] Roetzer A., Diel R., Kohl T. A. (2013). Whole genome sequencing versus traditional genotyping for investigation of a *Mycobacterium tuberculosis* outbreak: a longitudinal molecular epidemiological study. *PLoS Medicine*.

[38] Kanai M., Yeo T., Asua V., Rosenthal P. J., Fidock D. A., Mok S. (2022). Comparative analysis of *Plasmodium falciparum* genotyping via SNP detection, microsatellite profiling, and whole-genome sequencing. *Antimicrobial Agents and Chemotherapy*.

[39] Neafsey D. E., Taylor A. R., MacInnis B. L. (2021). Advances and opportunities in malaria population genomics. *Nature Reviews Genetics*.

[40] Leal S. D. V., Ward D., Campino S. (2021). Drug resistance profile and clonality of *Plasmodium falciparum* parasites in Cape Verde: the 2017 malaria outbreak. *Malaria Journal*.

[41] Wasakul V., Disratthakit A., Mayxay M. (2023). Malaria outbreak in Laos driven by a selective sweep for *Plasmodium falciparum kelch13* R539T mutants: a genetic epidemiology analysis. *Lancet Infectious Diseases*.

[42] Kittichai V., Koepfli C., Nguitragool W., Sattabongkot J., Cui L. (2017). Substantial population structure of *Plasmodium vivax* in Thailand facilitates identification of the sources of residual transmission. *PLoS Neglected Tropical Diseases*.

[43] Brasil P., Zalis M. G., de Pina-Costa A. (2017). Outbreak of human malaria caused by *Plasmodium simium* in the Atlantic Forest in Rio de Janeiro: a molecular epidemiological investigation. *Lancet Global Health*.

[44] Brashear A. M., Fan Q., Hu Y. (2020). Population genomics identifies a distinct *Plasmodium vivax* population on the China–Myanmar border of Southeast Asia. *PLoS Neglected Tropical Diseases*.

[45] MalariaGEN I. Adam, Alam M. S., Alemu S. (2022). An open dataset of *Plasmodium vivax* genome variation in 1,895 worldwide samples. *Wellcome Open Research*.

[46] Geng J., Malla P., Zhang J. (2019). Increasing trends of malaria in a border area of the Greater Mekong Subregion. *Malaria Journal*.

[47] Price M. N., Dehal P. S., Arkin A. P. (2010). FastTree 2—approximately maximum-likelihood trees for large alignments. *PLoS One*.

[48] Schaffner S. F., Taylor A. R., Wong W., Wirth D. F., Neafsey D. E. (2018). hmmIBD: software to infer pairwise identity by descent between haploid genotypes. *Malaria Journal*.

[49] Robertson L. J., Clark C. G., Debenham J. J. (2019). Are molecular tools clarifying or confusing our understanding of the public health threat from zoonotic enteric protozoa in wildlife?. *International Journal for Parasitology: Parasites and Wildlife*.

[50] Fernández-Escobar M., Schares G., Maksimov P., Joeres M., Ortega-Mora L. M., Calero-Bernal R. (2022). *Toxoplasma gondii* genotyping: a closer look into Europe. *Frontiers in Cellular and Infection Microbiology*.

[51] Sundararaman B., Shapiro K., Packham A. (2024). Whole genome enrichment approach for genomic surveillance of *Toxoplasma gondii*. *Food Microbiology*.

[52] Huang W., Guo Y., Lysen C. (2023). Multiple introductions and recombination events underlie the emergence of a hyper-transmissible *Cryptosporidium hominis* subtype in the USA. *Cell Host & Microbe*.

[53] Nader J. L., Mathers T. C., Ward B. J. (2019). Evolutionary genomics of anthroponosis in *Cryptosporidium*. *Nature Microbiology*.

[54] Xu Z., Li N., Guo Y., Feng Y., Xiao L. (2020). Comparative genomic analysis of three intestinal species reveals reductions in secreted pathogenesis determinants in bovine-specific and non-pathogenic *Cryptosporidium* species. *Microbial Genomics*.

[55] Li J., Li N., Roellig D. M. (2023). High subtelomeric GC content in the genome of a zoonotic *Cryptosporidium* species. *Microbial Genomics*.

[56] Su X.-Z., Lane K. D., Xia L., Sá J. M., Wellems T. E. (2019). *Plasmodium* genomics and genetics: new insights into malaria pathogenesis, drug resistance, epidemiology, and evolution. *Clinical Microbiology Reviews*.

[57] MalariaGEN M. M. A. H., Abdelraheem M. H., Acheampong D. O. (2023). Pf7: an open dataset of *Plasmodium falciparum* genome variation in 20,000 worldwide samples. *Wellcome Open Research*.

[58] MalariaGEN A. A., Ali M., Almagro-Garcia J. (2021). An open dataset of *Plasmodium falciparum* genome variation in 7,000 worldwide samples. *Wellcome Open Research*.

[59] Taylor A. R., Schaffner S. F., Cerqueira G. C. (2017). Quantifying connectivity between local *Plasmodium falciparum* malaria parasite populations using identity by descent. *PLoS Genetics*.

[60] Tessema S. K., Raman J., Duffy C. W., Ishengoma D. S., Amambua-Ngwa A., Greenhouse B. (2019). Applying next-generation sequencing to track *falciparum* malaria in sub-Saharan Africa. *Malaria Journal*.

[61] Lorenzi H., Khan A., Behnke M. S. (2016). Local admixture of amplified and diversified secreted pathogenesis determinants shapes mosaic *Toxoplasma gondii* genomes. *Nature Communications*.

[62] Galal L., Ariey F., Gouilh M. A. (2022). A unique *Toxoplasma gondii* haplotype accompanied the global expansion of cats. *Nature Communications*.

[63] Wang T., Guo Y., Roellig D. M. (2022). Sympatric recombination in zoonotic *Cryptosporidium* leads to emergence of populations with modified host preference. *Molecular Biology and Evolution*.

[64] Corsi G. I., Tichkule S., Sannella A. R. (2023). Recent genetic exchanges and admixture shape the genome and population structure of the zoonotic pathogen *Cryptosporidium parvum*. *Molecular Ecology*.

[65] Tichkule S., Cacciò S. M., Robinson G. (2022). Global population genomics of two subspecies of *Cryptosporidium hominis* during 500 years of evolution. *Molecular Biology and Evolution*.

[66] Troell K., Hallström B., Divne A.-M. (2016). *Cryptosporidium* as a testbed for single cell genome characterization of unicellular eukaryotes. *BMC Genomics*.

[67] Manske M., Miotto O., Campino S. (2012). Analysis of *Plasmodium falciparum* diversity in natural infections by deep sequencing. *Nature*.

[68] Park D. J., Lukens A. K., Neafsey D. E. (2012). Sequence-based association and selection scans identify drug resistance loci in the *Plasmodium falciparum* malaria parasite. *Proceedings of the National Academy of Sciences of the United States of America*.

[69] Mobegi V. A., Duffy C. W., Amambua-Ngwa A. (2014). Genome-wide analysis of selection on the malaria parasite *Plasmodium falciparum* in West African populations of differing infection endemicity. *Molecular Biology and Evolution*.

[70] Deelder W., Benavente E. D., Phelan J. (2021). Using deep learning to identify recent positive selection in malaria parasite sequence data. *Malaria Journal*.

[71] Camponovo F., Buckee C. O., Taylor A. R. (2023). Measurably recombining malaria parasites. *Trends in Parasitology*.

[72] Hamilton W. L., Amato R., v. d. Pluijm R. W. (2019). Evolution and expansion of multidrug-resistant malaria in southeast Asia: a genomic epidemiology study. *Lancet Infectious Diseases*.

[73] Lefebvre M. J. M., Daron J., Legrand E., Fontaine M. C., Rougeron V., Prugnolle F. (2023). Population genomic evidence of adaptive response during the invasion history of *Plasmodium falciparum* in the Americas. *Molecular Biology and Evolution*.

[74] Taylor A. R., Echeverry D. F., Anderson T. J. C., Neafsey D. E., Buckee C. O. (2020). Identity-by-descent with uncertainty characterises connectivity of *Plasmodium falciparum* populations on the Colombian-Pacific coast. *PLoS Genetics*.

[75] Wong W., Griggs A. D., Daniels R. F. (2017). Genetic relatedness analysis reveals the cotransmission of genetically related *Plasmodium falciparum* parasites in Thies, Senegal. *Genome Medicine*.

[76] Divis P. C. S., Duffy C. W., Kadir K. A., Singh B., Conway D. J. (2018). Genome-wide mosaicism in divergence between zoonotic malaria parasite subpopulations with separate sympatric transmission cycles. *Molecular Ecology*.

[77] Hupalo D. N., Luo Z., Melnikov A. (2016). Population genomics studies identify signatures of global dispersal and drug resistance in *Plasmodium vivax*. *Nature Genetics*.

[78] Daron J., Boissière A., Boundenga L. (2021). Population genomic evidence of *Plasmodium vivax* Southeast Asian origin. *Science Advances*.

[79] d. Oliveira T. C., Rodrigues P. T., Early A. M. (2021). *Plasmodium simium*: population genomics reveals the origin of a reverse zoonosis. *Journal of Infectious Diseases*.

[80] Trimarsanto H., Amato R., Pearson R. D. (2022). A molecular barcode and web-based data analysis tool to identify imported *Plasmodium vivax* malaria. *Communications Biology*.

[81] Wilson B. A., Garud N. R., Feder A. F., Assaf Z. J., Pennings P. S. (2016). The population genetics of drug resistance evolution in natural populations of viral, bacterial and eukaryotic pathogens. *Molecular Ecology*.

[82] Ahuir-Baraja A. E., Cibot F., Llobat L., Garijo M. M. (2021). Anthelmintic resistance: is a solution possible?. *Experimental Parasitology*.

[83] Warhurst D. C. (2001). A molecular marker for chloroquine-resistant falciparum malaria. *New England Journal of Medicine*.

[84] Pelleau S., Moss E. L., Dhingra S. K. (2015). Adaptive evolution of malaria parasites in French Guiana: reversal of chloroquine resistance by acquisition of a mutation in *pfcrt*. *Proceedings of the National Academy of Sciences of the United States of America*.

[85] Amambua-Ngwa A., Button-Simons K. A., Li X. (2023). Chloroquine resistance evolution in *Plasmodium falciparum* is mediated by the putative amino acid transporter AAT1. *Nature Microbiology*.

[86] Auburn S., Benavente E. D., Miotto O. (2018). Genomic analysis of a pre-elimination Malaysian *Plasmodium vivax* population reveals selective pressures and changing transmission dynamics. *Nature Communications*.

[87] Price R. N., Auburn S., Marfurt J., Cheng Q. (2012). Phenotypic and genotypic characterisation of drug-resistant *Plasmodium vivax*. *Trends in Parasitology*.

[88] Sá J. M., Kaslow S. R., Barros R. R. M. (2019). *Plasmodium vivax* chloroquine resistance links to pvcrt transcription in a genetic cross. *Nature Communications*.

[89] Taylor S., Barragan A., Su C. (2006). A secreted serine-threonine kinase determines virulence in the eukaryotic pathogen *Toxoplasma gondii*. *Science*.

[90] Reid A. J., Vermont S. J., Cotton J. A. (2012). Comparative genomics of the apicomplexan parasites *Toxoplasma gondii* and *Neospora caninum*: Coccidia differing in host range and transmission strategy. *PLoS Pathogens*.

[91] Feng Y., Li N., Roellig D. M. (2017). Comparative genomic analysis of the IId subtype family of *Cryptosporidium parvum*. *International Journal for Parasitology*.

[92] Auburn S., Getachew S., Pearson R. D. (2019). Genomic analysis of *Plasmodium vivax* in southern Ethiopia reveals selective pressures in multiple parasite mechanisms. *Journal of Infectious Diseases*.

[93] Mourier T., d. Alvarenga D. A. M., Kaushik A. (2021). The genome of the zoonotic malaria parasite *Plasmodium simium* reveals adaptations to host switching. *BMC Biology*.

[94] Su X.-z., Heatwole V. M., Wertheimer S. P. (1995). The large diverse gene family var encodes proteins involved in cytoadherence and antigenic variation of *Plasmodium falciparum*-infected erythrocytes. *Cell*.

[95] del Portillo H. A., Fernandez-Becerra C., Bowman S. (2001). A superfamily of variant genes encoded in the subtelomeric region of *Plasmodium vivax*. *Nature*.

[96] Berriman M., Ghedin E., Hertz-Fowler C. (2005). The genome of the African trypanosome *Trypanosoma brucei*. *Science*.

[97] Vollger M. R., Logsdon G. A., Audano P. A. (2020). Improved assembly and variant detection of a haploid human genome using single-molecule, high-fidelity long reads. *Annals of Human Genetics*.

[98] Lapp S. A., Geraldo J. A., Chien J.-T. (2018). PacBio assembly of a *Plasmodium knowlesi* genome sequence with Hi-C correction and manual annotation of the *SICAvar* gene family. *Parasitology*.

[99] Ueti M. W., Johnson W. C., Kappmeyer L. S. (2021). Comparative analysis of gene expression between *Babesia bovis* blood stages and kinetes allowed by improved genome annotation. *International Journal for Parasitology*.

[100] Nag S., Kofoed P.-E., Ursing J. (2018). Direct whole-genome sequencing of *Plasmodium falciparum* specimens from dried erythrocyte spots. *Malaria Journal*.

[101] Domagalska M. A., Imamura H., Sanders M. (2019). Genomes of *Leishmania* parasites directly sequenced from patients with visceral leishmaniasis in the Indian subcontinent. *PLoS Neglected Tropical Diseases*.

[102] Schneeberger P. H. H., Becker S. L., Pothier J. F. (2016). Metagenomic diagnostics for the simultaneous detection of multiple pathogens in human stool specimens from Cote d’Ivoire: a proof-of-concept study. *Infection Genetics and Evolution*.

[103] Deelder W., Manko E., Phelan J. E., Campino S., Palla L., Clark T. G. (2022). Geographical classification of malaria parasites through applying machine learning to whole genome sequence data. *Scientific Reports*.

[104] Hu R.-S., Hesham A. E.-L., Zou Q. (2022). Machine learning and its applications for protozoal pathogens and protozoal infectious diseases. *Frontiers in Cellular and Infection Microbiology*.

[105] Zheng X., Levine D., Shen J., Gogarten S. M., Laurie C., Weir B. S. (2012). A high-performance computing toolset for relatedness and principal component analysis of SNP data. *Bioinformatics*.

[106] Raj A., Stephens M., Pritchard J. K. (2014). fastSTRUCTURE: variational inference of population structure in large SNP data sets. *Genetics*.

[107] Huson D. H., Bryant D. (2006). Application of phylogenetic networks in evolutionary studies. *Molecular Biology and Evolution*.

[108] Ward B. J., v. Oosterhout C. (2016). HYBRIDCHECK: software for the rapid detection, visualization and dating of recombinant regions in genome sequence data. *Molecular Ecology Resources*.

[109] Malinsky M., Matschiner M., Svardal H. (2021). Dsuite - Fast *D*-statistics and related admixture evidence from VCF files. *Molecular Ecology Resources*.

[110] Alexander D. H., Novembre J., Lange K. (2009). Fast model-based estimation of ancestry in unrelated individuals. *Genome Research*.

